# The gut bacterial diversity of sheep associated with different breeds in Qinghai province

**DOI:** 10.1186/s12917-020-02477-2

**Published:** 2020-07-23

**Authors:** Jianjun Chang, Xiaoting Yao, Chenxiang Zuo, Yuxu Qi, Dekun Chen, Wentao Ma

**Affiliations:** 1 State Key Laboratory of Plateau Ecology and Agriculture, Qinghai University, Xining, 810016 Qinghai Province China; 2 College of Agriculture and Animal Husbandry, Qinghai University, Xining, 810016 Qinghai Province China; 3 College of Veterinary Medicine, Northwest A&F University, Yangling, 712100 Shaanxi Province China

**Keywords:** Tibetan sheep, Gut microbiota, High throughput sequencing, Breeds diversity

## Abstract

**Background:**

Gut microbiota play important roles in their co-evolution with mammals. However, little is understood about gut bacterial community of Tibetan sheep compared with other sheep breeds. In this study, we investigated the gut bacterial community in 4 different sheep breeds living in the Qinghai-Tibetan Plateau (QTP) of China using high-throughput sequencing (HTS) technique.

**Results:**

The results suggested that bacterial community abundance and breeds diversity of Tibetan sheep (TS) were significantly lower than that of the other three breeds of sheep [Dorset sheep (DrS), Dorper sheep (DrS) and Small Tail Han sheep (STHS)] (*p* < 0.05). Principal coordinates analysis (PCoA) and nonmetric multidimensional scaling (NMDS) analysis indicated that microbiome composition of TS was significantly different from that of other three sheep breeds (*p* < 0.01). *Firmicutes* was the most predominant microbial phylum in the gut, followed by *Bacteroidetes*. The gut bacterial community of TS showed higher proportions of phylum *Spirochaetes*, *Proteobacteria* and *Verrucomicrobia*, compared to the other three sheep breeds, but the *Deferribacteres* was absent in TS. At the genus level, *Treponema*, *Succinivibrio*, 5-7 N15 and *Prevotella* showed significantly higher abundance in TS than in the other three sheep breeds (*p* < 0.05).

**Conclusions:**

In this study, we first employed HTS to understand the gut microbiomes among different sheep breeds in QTP of China.

## Background

The significance of gut microbiome is well known, it is an extremely complicated and diverse population and has been explored extensively [1, 2]. Recently, a new term ‘superorganism’ is applied to describe the strong tie between the gut commensal and its reliable host [3, 4]. The intestinal microbiota is linked to a diverse range of conditions, including gathering energy, promoting intestinal epithelial cell proliferation and enhancing the immune system [5, 6], and can be deservedly regarded as an ‘organ’ playing a significant part in the metabolic process [3, 7, 8]. It has been found that more than 3.3 million commensal genes are resided in humans, which is equivalent to 150 times of human genes [9]. In ruminants, the composition of the gastrointestinal commensals, their effect on host immunity and welfare have been explored for several years. A previous study suggested that it was a great achievement of using gut microbiota by ruminant animals [10]. And it’s true that combining high-throughput ‘omics’ technologies with ruminant’s genomes, the unprecedented well-being of achievement can be harvested [3].

Initially, the studies about gut microorganisms were dependent on the sequence alignment from genomic libraries to screen the functional genes, or through PCR amplification [3]. In *Escherichia coli*, heterologous gene expression was determined by Sanger Chain Termination Method and obtained the autoradiographic map [11]. However, it could only obtain the genes expressed in *E. coli*, and certainly leads to a large missing of available genes. Through this method, the first gene was acquired from *F. succinogenes* and encoding cellulases [12]. In the following years, seven *F. succinogenes* genes were detected through these traditional genetic methods, which encoded fiber-degrading enzymes [13]. This was certainly an extraordinary progress, but after the sequencing of *F. succinogenes* S85 genome was completed, it was found that there were 104 open reading frames to participate in the disruption of plant cell wall [14]. Accordingly, the great advantage of genomic sequencing is intuitively clear, more enzymes were detected than that of previous studies in the *F. succinogenes* genome.

Metagenomics is performed to analyze the genome of microbial communities from an environmental sample, including the genomic sequence-based analysis and functional prediction. It is applied to screen the specific functions and detecting new bio-actives in diverse ecosystems [15]. Moreover, it is a vital step to model and connect the microbial structure and function to that of the host [16, 17]. Some researchers found that the gut microbiota of mammals have a large identical part of their functions, suggesting that the understanding of human researches can provide many common views for ruminants [18]. In addition, relationship between microbial lineages and their specific environment was found [19]. However, those gut microbes are not frequently emerged in other environments [20]. Some studies also showed that gut microbes were extensively shared among various mammals [21], indicating that some views are commonly applied to both humans and domestic animals.

In the present study, to better analyze and clarify the relationship between microbial lineages and the host breeds from a perspective of gut microbiota, V3 and V4 region of the 16 s rDNA was amplified, which was followed by, the most credible techniques, Illumina MiSeq Reagent Kit PE250 sequencing. Apart from the shared characteristics are presented among all sheep, typical microbial population features corresponding to different sheep breeds are also mentioned in our research.

## Methods

### Description of samples

A total of 40 sheep (male; 1 year old) with a similar feeding pattern were used in this research. Fecal samples including 10 Dorset sheep (DrS), 15 Small Tail Han sheep (STHS), 5 Tibetan sheep (TS) and 10 Dorper sheep (DrS) were obtained from specific farms (Qinghai Province, China). 16S rDNA sequences from 40 individuals were amplified and analyzed. All screened sheep were healthy and no other diseases appeared prior to the sample selection. Table S1 provides the detailed information of each sheep we sampled.

### DNA extraction

Following the manufacturer’s instructions of Omega Bio-tek, microbial genomic DNA was extracted from 500 mg of each fecal sample using the fecal DNA kit. Meanwhile, we measured the DNA quality with 1% agarose gel electrophoresis, and examined the concentration through the NanoDrop Spectrophotometer. DNA samples were stored at − 20 °C before use. The experimenters conducting the rDNA extraction of analysis were blinded to the group samples they were testing.

### PCR amplification of 16 s rDNA

The V3-V4 region, a 468 bp within the 16 s rDNA gene, was used to build the illumine sequencing library and amplified with the broadly conserved primers 341F (5′–CCTACGGGNGGCWGCAG-3′) and 805R (5′-GACTACHVGGGTATCTAATCC-3′). Different identifier codes were added at each primer for the further illumina sequencing. Polymerase chain reaction (PCR) was applied in a 50 μl reaction system including 25 μl 2x Phanta Max Master Mix (Vazyme, China), 10 mM each primer, 16 μl each ddH2O and 5 μl DNA template. The PCR program was initial denaturation at 95 °C, with 8 cycles of denaturation at 95 °C for 30s, annealing at 55 °C for 30 s, extension at 72 °C for 45 s, with a final elongation phase at 72 °C for 5 min. The PCR products were analyzed by Quant-It Pico Green kit (Invitrogen, United States) and library was prepared. Barcoded samples were combined equal concentrations according to volume of sequencing. The library concentration was measured using Agilent 2100 Bioanalyzer (Agilent Technologies, United States), and followed by elution with Tris_HCl (pH 8.5). After denaturation, barcoded samples were combined following the volume of sequencing and sequenced on a PE250 v3 instrument using 600 cycles MiSeq Reagent Kit on a MiSeq Platform (Illumina; United States).

### Bioinformatics and statistical analysis

In our research, all sequences have been deposited to the National Center for Biotechnology Information (NCBI) database under accession number AR180907. The QIIME (Quantitative Insights Into Microbial Ecology, v1.8.0) was performed to process the raw reads, and then the paired reads were assembled by FLASH v1.2.7 [22, 23]. Subsequently, QIIME was used to filter and analyze the joined sequences. By UPARSE 7.0, operational taxonomic units (OTUs) were obtained with based on a 97% identity threshold. Eventually, the whole OTUs were categorized to distinct taxonomic levels by Ribosomal Database Project (RDP) classifier 2.2 [24]. Based on the OTUs information, R package VennDiagram was performed to complete the venn diagram. In addition, the phylogenetic tree was obtained by MAGA 5.2 after sequences alignment. Alpha diversity was measured by MOTHUR, which was referred to the microbial community diversity. Bray-Curtis distance and unweighted Unifrac was evaluated the similarities of different samples with R package vegan. The Bray-Curtis distance was estimated based on common OTUs among samples to provide equal weight to differences in each taxa [1, 25]. The Unifrac was used to construct the phylogenetic tree for samples. Taxa which are phylogenetically closely related will give less divergent Unifrac values, while the unrelated taxa will generate larger values [26]. OIIME was performed to generate phylogenetic beta diversity. Principal coordinate analysis (PCoA) and hierarchical clustering analysis by R program was conducted based on Bray-Curtis distance and unweighted Unifrac. PERMANOVA and student’s t-test were performed to exam significant differences between various groups.

## Results

### Description of the sequencing data

Fecal samples were collected from four different sheep breeds (10 Dorset sheep (DrS), 15 Small Tail Han sheep (STHS), 5 Tibetan sheep (TS) and 10 Dorper sheep (DrS)). We retrieved 1,694,264 raw bases from the sequencing platform, as mentioned above. After quality–filtering (also as described in the methods), 1,359,405 total sequences with an average of 433 bp in length were obtained for the following analysis.

### Gut microbiota is associated with sheep breeds

**Fig. 1:**
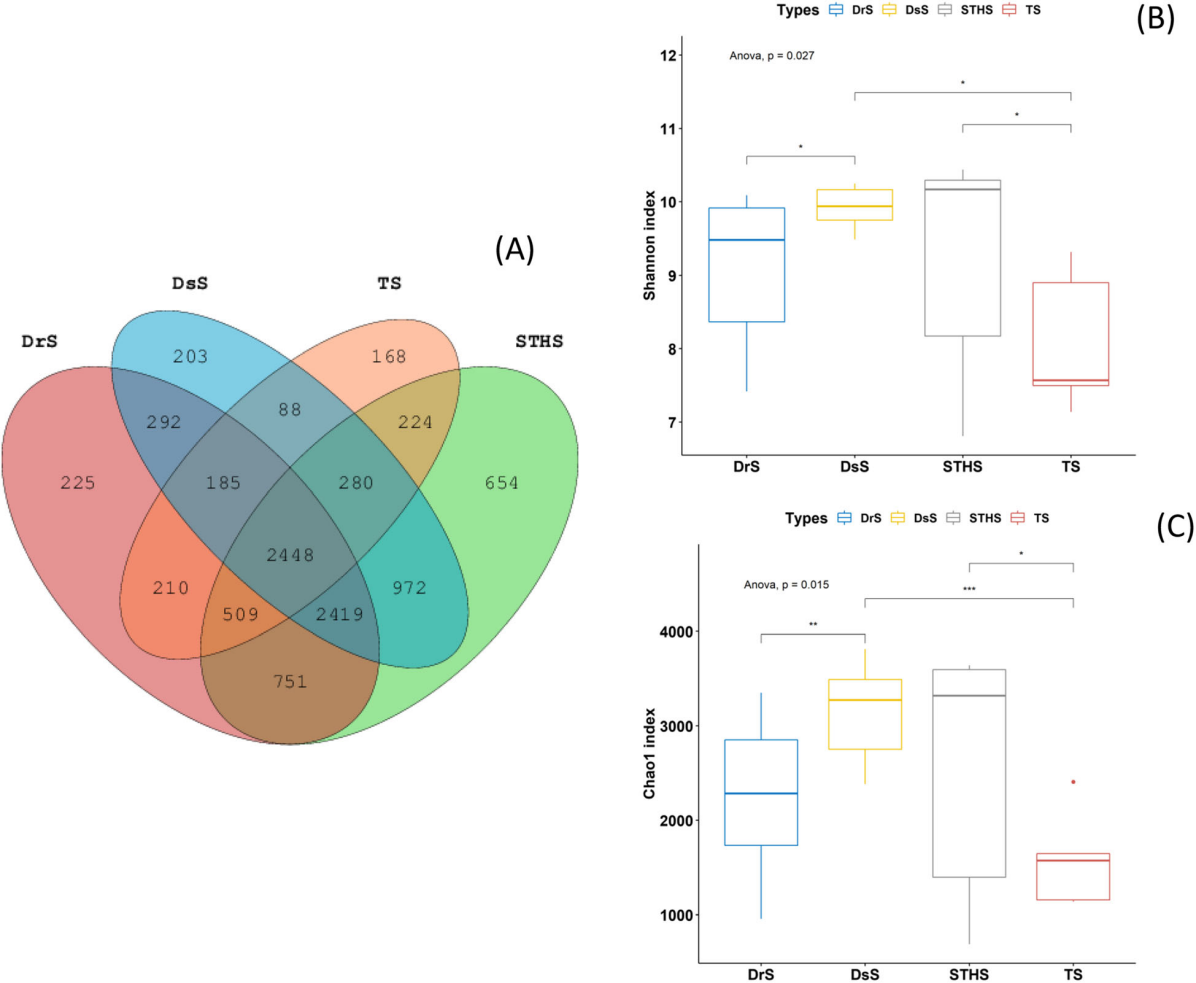
The community composition and microbial diversity index analysis. **a** Venn diagram showing overlap in OTUs of differential abundance in DrS, DsS, TS and STHS. **b** Shannon index. **c** Chao1 index. Different asterisks represent statistical significance (*p < 0.05, ** *p* < 0.01, *** *p* < 0.001)

After cleaning the original data, 1,359,405 high-quality available sequences were obtained. According to 97% breeds similarity, 7039, 6887, 4112, and 8257 OTUs were acquired from samples at groups DrS, DsS, TS, and STHS (Table S2), respectively. A total of 26,295 OTUs were detected from all samples, of which 2448 were core OTUs (Fig. 1a). The core OTUs accounts for nearly 9.31% of the entire OTUs. Furthermore, 225,203 OTUs were uniquely detected in DrS and DsS groups, and 168,654 unique OTUs were found in group TS and STHS, respectively. The number of observed OTUs in the TS samples was fewer than that of the other three breeds.

To confirm the quality of our sequencing data, we examined alpha and beta diversities of bacterial fraction of the sheep microbiota. Several alpha diversity indices diverged significantly between the four sheep breeds (Fig. 1b and c). The Shannon-Wiener index may straightly indicate the heterogeneity of a community according to the OTU counts of breeds and their related abundance [27]. The Shannon-Wiener indicator of groups DsS, STHS, TS and DrS were 9.92, 9.33, 8.09 and 9.06, respectively. Within the groups, Chao1 and Shannon index visually reflected that the abundance and diversity of intestinal microbial population in the TS group were lower than those in groups DsS, STHS and DrS, and the difference among these four groups was significant (*P* < 0.05; Fig. 1b and c). The Chao1 index of DsS, STHS, TS and DrS breeds was 3160, 2598, 1585 and 2207 respectively. The ACE indices of these four breeds were 3263, 2646, 1627 and 2267 (Table S3), which was consistent with the Chao1 results, suggesting that the OTU richness of TS samples was lower than other three breeds (Fig. 1b and c). Collectively, these data pointed towards a more diverse bacterial population in TS compared to others, and also showed that differences in intestinal microbial composition associates sheep breeds.

### Comparison of bacterial microbiome diversity among different sheep breeds

**Fig. 2:**
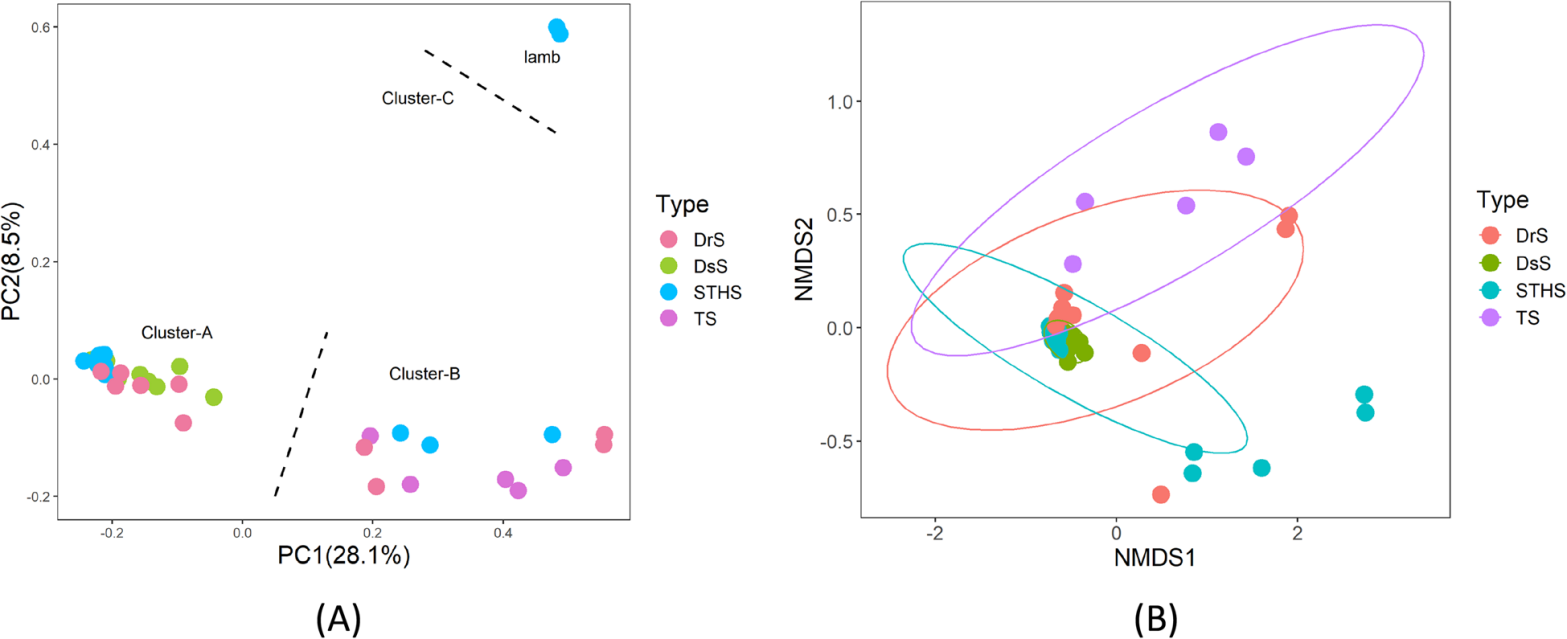
Compositional analyses of the gut microbiome of different sheep breeds. **a** PCoA plot of similarities between the different groups. Principal component (PC) 1 and 2 accounted for 28.1 and 8.5% of the variance, respectively. **b** NMDS showing the alteration of bacterial population based on Bray-Curtis distance

The Bray-Curtis distance matrices were measured according to the OTUs abundance of each sample. Based on the distance matrices, the unweighted Unifrac similarity analysis indicated that the similarities among different sheep breeds were significant. The principal coordinates analysis (PCoA) was performed according to the phylogenetic-tree-based Unifrac metric. As shown in Fig. 2a, samples were sequestered into three clusters. Scattered points in the principal component denoted different breeds and their relationship between each other. There were significant differences among breeds in relation to microbiome composition (PERMANOVA, *p* < 0.01). TS were mainly aggregated in cluster B, whereas DsS were mostly converged in cluster A. Moreover, DrS and STHS were more scattered and found between cluster A and cluster B. Both principal components accounted for 28.1% (PC1) and 8.5% (PC2) of the explained variance. Interestingly, two lambs in STHS were clustered separately as shown in Fig. 2a, suggesting that there was a general difference in gut microbiome between adult sheep and lambs.

Nonmetric multidimensional scaling (NMDS) was used to further clarify the difference among the bacterial population of all breeds, which was performed using the Bray-Curtis similarity for all samples at OTU level [1]. As a dominant ordination method that could exhibit the non-linear relationship among samples, NMDS has been widely applied in the study of gut microbiome. As shown in Fig. 2b, there was distinguishing clustering of TS samples, meanwhile samples from DsS were very close to DrS. However, the samples from STHS were more dispersed (Fig. 2b).

**Fig. 3:**
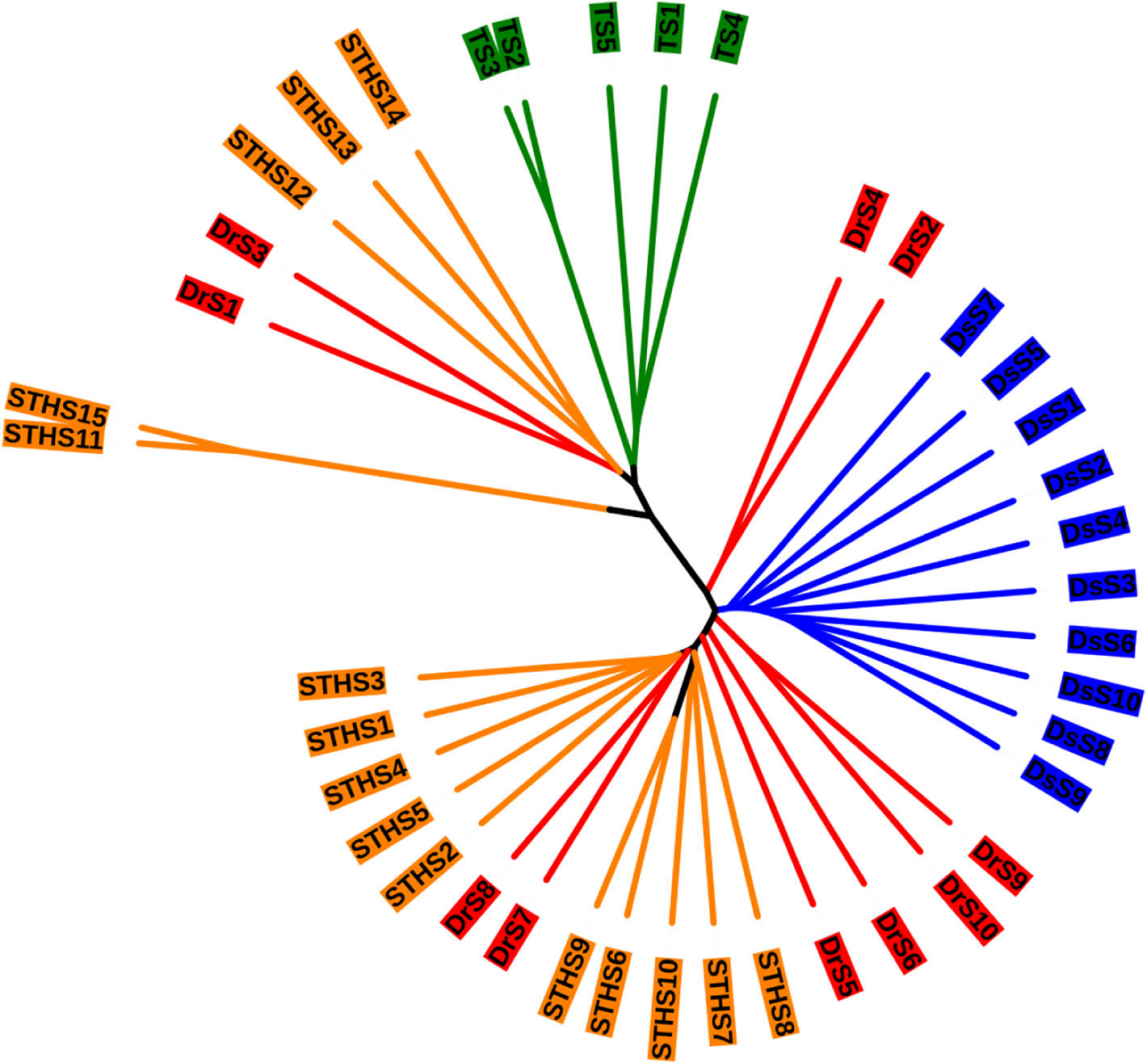
Hierarchical clustering of bacterial communities according to Bray-Curtis distance

Additionally, we did hierarchical clustering analysis of all samples to exhibit the similarity among samples, which was performed with Unweighted pair-group method with arithmetic means (UPGMA) and the Bray-Curtis similarity. Two primary groups were perceived in this analysis. One cluster contains all TS samples and the other cluster contains all samples from DrS (Fig. 3). Consist with the results above, TS samples were distinctive compared with other breeds. In general, the composition of gut microbiota is greatly influenced by the breed of sheep.

### Gut microbial diversities and community composition among different sheep breeds

**Fig. 4:**
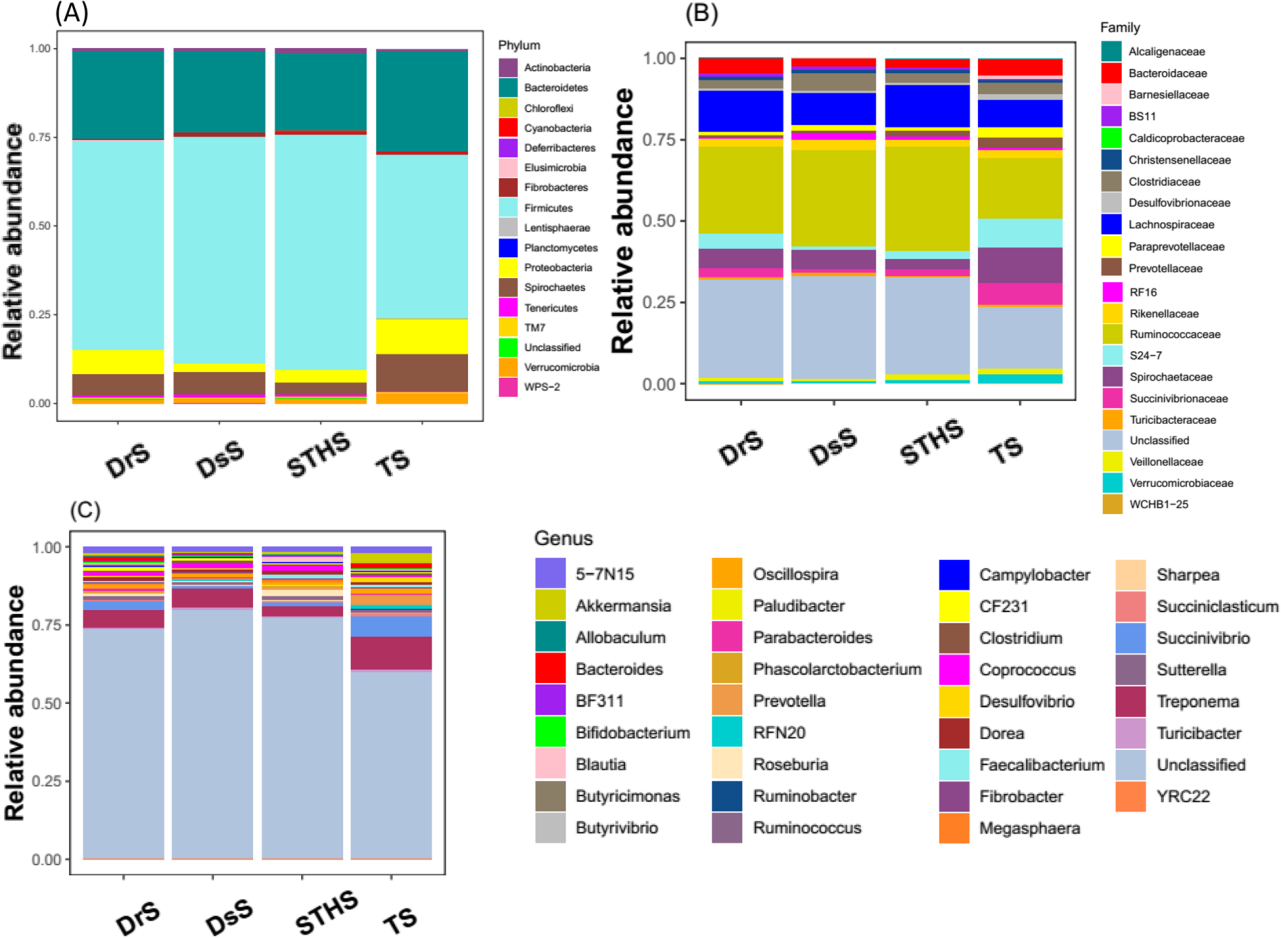
Microbial composition of different samples. Each bar represents the average relative abundance of each bacterial taxon within a group. **a** Taxa assignments at Phylum level. **b** Taxa assignments at Family level. **c** Taxa assignments at Genus level

In order to clarify the diversity of gut bacterial composition in different sheep breeds, we estimated the gut microbiota in different taxonomical levels. The overall bacterial composition of different groups at the phylum level is illustrated in Fig. 4a, which shows that *Firmicutes* was the most predominant phylum in all samples, followed by *Bacteroidetes*. Higher abundance of phylum *Spirochaetes*, *Proteobacteria* and *Verrucomicrobia* was found in TS than those in other three breeds, but the *Deferribacteres* was absent in TS (Fig. 4a).

When analyzed on the family level, as shown in Fig. 4b, no significant differences were detected among these four groups. *Ruminococcaceae* and *WCHB1–25* were the most abundant families in DsS, DrS and STHS group, whereas *Alcaligenaceae*, *Desulfovibrionaceae* and *Barnesiellaceae* were almost absent. As for TS group, the most abundant families were *Spirochaetaceae*, *S24–7*, *Prevotellaceae*, *Barnesiellaceae* and *Succinivibrionaceae*, while BS11 and WCHB1–25 were almost absent in the TS samples (Fig. 4b).

In contrast to the family level, there are significant differences between TS group and the other three groups on the genus level. The main genera in TS group included *Treponema*, *Succinivibrio*, 5-7 N15 and *Prevotella* (Table 1), while *Bifidobacterium*, *Sharpea* and *YRC22* were absent (Fig. 4c). Moreover, in DsS, STHS and DrS group, *Treponema* remained the predominant population, and *Coprococcus* and *Roseburia* were relatively less abundant. However, it is worth mentioning that a large number of microbes in TS samples were relatively abundant, when compared to other three groups.

**Table 1:** The relative abundance of top 10 genera bacteria in four sheep breeds, expressed as an average percentage of the total (*n* = 40)

Genera	Group				Class	Phylum
	DsS	STHS	TS	DrS		
Treponema	6.11	3.09	10.63	5.90	Spirochaetes	Spirochaetes
Succinivibrio	0.82	1.15	6.52	2.96	Gammaproteobacteria	Proteobacteria
5-7N15	1.36	1.35	1.91	1.84	Bacteroidia	Bacteroidetes
Akkermansia	0.70	1.06	2.95	0.87	Verrucomicrobiae	Verrucomicrobia
Coprococcus	1.66	1.36	0.79	1.60	Clostridia	Firmicutes
Oscillospira	1.05	1.19	1.49	1.22	Clostridia	Firmicutes
Ruminococcus	0.87	1.33	0.86	1.25	Clostridia	Firmicutes
Prevotella	0.29	0.83	2.82	0.35	Bacteroidia	Bacteroidetes
Dorea	0.79	1.12	0.79	1.20	Clostridia	Firmicutes
Bacteroides	0.14	0.22	1.80	1.58	Bacteroidia	Bacteroidetes
Other	86.21	87.3	69.44	81.23		

## Discussion

This study was aimed at acquiring insight into the gut bacterial composition of four sheep breeds living in the QTP of China using next-generation sequencing technique. In our study, the gut bacterial community of four different sheep breeds was estimated by PCR-retrieved microbial 16S rDNA gene libraries. In our research, intestinal microflora of four sheep breeds has been examined by bacterial diversity and abundance. These results indicated that there are significant differences of the gut microbiota between TS and the other three sheep breeds (DrS, DsS and STHS), besides, the bacterial diversity and composition of TS are relatively lower. However, the bacterial abundance in TS are higher than those in the other three sheep breeds. The microbial diversity of TS altered significantly compared with the other three breeds, which is similar with the earlier reports in high-altitude mammals [28, 29]. PCoA clustering analysis revealed that the microbial structure is distinct between the TS and the other three breeds (Fig. 2a). Besides, hierarchical clustering analysis showed that TS samples clearly cluster together, indicating that the intestinal microbial population of TS are highly conserved for the comparison between interbreeds. On different taxonomical levels, the abundance of gut bacterial composition is also distinct among different sheep breeds. This phenomenon is probably due to the fact that Tibetan sheep have adapted to the high-altitude environment, while the other three breeds, as introduced later, are convergent to the commensal composition of Tibetan sheep. In addition, our study showed that the gut bacterial composition of lambs is quite different from that in adult sheep. We assumed that the gut microbial composition in lambs will develop towards adult sheep under their living environment.

This study suggested that *Firmicutes* and *Bacteroidetes* are the most abundant phyla in the gut microbiomes of all samples, which accounted for 55.83 and 24.39% of the total microbial abundance, respectively, which was consistent with the prior studies in herbivores [30]. The functions of *Firmicutes* and *Bacteroidetes* are closely associated with carbohydrate, protein, and fiber metabolism [31]. The crop straws mainly contained cellulose, hemicellulose and lignin, and the *Firmicutes* contained a lot of fiber-decomposing bacteria, including *Butyvibrio*, *Ruminococcus*, *Oscillibacter* and *Eubacterium*, which may explain why the *Firmicutes* are dominant in the rumen bacterial community of ruminants [30]. *Bacteroidetes* are the major degraders for decomposing non-fibrous plant components in sheep gut, and the *Prevotella* have the highest composition in this bacteria group. It has been suggested that *Prevotella* can account for 60–70% of the overall microbial communities in rumen, and it includes highly active hemicellulose decomposing microbes, which was important for the decomposition of non-fibrous polysaccharides or proteins in crops [32, 33].

In this study, the relative abundance of *Firmicutes* and *Bacteroidetes* were not consistent among the different sheep breeds, *Bacteroidetes* had higher abundance in TS than the other three breeds. In order to investigate the reasons of this result, we estimated the genera level of taxonomy and found that within the phylum *Bacteroidetes*, three genera had significantly higher abundance in TS than the other three sheep breeds, including *5-7 N15*, *Prevotella* and *Bacteroides*. These three genera belong to the class *Bacteroidia* (Table 1). Previous study has shown that the *Prevotella* played an important influence on the fermentation process of feed in the rumen of sika deer [34]. Other studies also suggested that *Prevotella* was predominant in ruminants [33, 35, 36]. *Prevotella* was used to degrade lignocellulosic feedstock with xylanase and carboxymethylcellulase [37]. Besides, some genera of the class *Bacteroidia* had high active hemicellulose decomposition which provides hosts with the abilities to digest and extract nutrition from fibrous plants [38]. In our research, we hypothesized that this phenomenon is probably due to the fact that TS have unique microbiota structure which was adapted to the high-altitude environment, while the other three breeds TS share greater similarity.

A special bacterial phylum called *Proteobacteria* was identified in the four breeds, which was observed in various ruminants, such as sheep and cattle. But a relative low proportion (0.8%) was found in yaks [39–41]. In this study, *Proteobacteria* had significantly higher abundance in TS than the other three breeds (*p* < 0.01). It has been reported that phylum *Proteobacteria* had the highest richness in the bovine rumen [42], and played a crucial role in the biofilm formation, fermentation and the soluble carbohydrates digestion [43]. We inferred that the reason for the higher abundance of *Proteobacteria* in TS was related to the different breeds of sheep. TS have adapted to survive in this harsh plateau environment, where Tibetans raise these animals for food and sustenance [28, 44]. Several studies revealed that the gut microbes of ruminants help them survival at high altitude, which involve in the energy metabolism pathway [28, 29, 45].

A large body of evidence has shown that gut microbial community composition is affected by animal breed and several other factors. For example, a study on Yak and Tibetan sheep demonstrated that the difference in bacterial compositions of the gut was mainly attributed to host breed [46]. Similarly, a report about the gut microbiota of *Bos taurus* and *Bubalus bubalis* demonstrated that rumen microbiota community varied with both breeds and feeding patterns [47]. Consistent with these studies, our results suggested that sheep breeds may critically determine their gut bacterial community. Other factors influencing gut microbiota structure includes geographical environment, which especially affects the richness of *Prevotellaceae*, *Butyrivibrio*, and *Campylobacter* [48]. Meanwhile, feeding regimens not only affect gut microbial composition, but also influence metabolic homeostasis in sheep [49, 50]. Another study also demonstrated that feeding with rosemary leaves could alter the abundance of rumen microbial community into one that was dominant with bacterial species involved in protein degradation and methane production [51].

## Conclusion

To conclude, our 16S rDNA analyses reflected the gut microbiome of different sheep breeds and highlighted the difference between TS and the other three sheep breeds (DsS, STHS and DrS). This study also showed a lot of high abundance species, which may play important roles in the host. The fluctuation of gut bacteria composition indicated that gut microbes could be changed along with the differences of sheep breeds. Different breeds caused a shift on the microbial community structure and decreased the bacterial species diversity in the gut of TS. Furthermore, the investigation of these distinct microbial structures may provide a better understanding for the light on mechanisms of these comparatively abundant, but enigmatic microbial symbionts of ruminants.

## Supplementary information

**Additional file 1: Table S1.** The information of sheep in this study. **Table S2.** Sequence data of samples. **Table S3.** The estimators of sequence diversity and richness.

## Data Availability

All data generated or analyzed during this study are included in this published article, and also available from the corresponding author on reasonable request.

## Abbreviations

DrS: Dorset sheep
STHS: Small Tail Han sheep
TS: Tibetan sheep
DrS: Dorper sheep
NCBI: National Center for Biotechnology Information
QIIME: Quantitative Insights Into Microbial Ecology
OTU: Operational taxonomic units
RDP: Ribosomal Database Project
PCoA: Principal coordinate analysis
PICRUSt: Phylogenetic Investigation of Communities by Reconstruction of Unobserved States
NMDS: Nonmetric multidimensional scaling
UPGMA: Unweighted pair-group method with arithmetic means
QTP: Qinghai-Tibetan Plateau
