# Supplementary material for: The gut bacterial diversity of sheep associated with different breeds in Qinghai province
Source: BMC Vet Res. 2020 Jul 23;16:254. doi: 10.1186/s12917-020-02477-2 (PMC7376942; doi:10.1186/s12917-020-02477-2)
Supplement: Supplementary file 1 — Additional file 1: Table S1. The information of sheep in this study. Table S2. Sequence data of samples. Table S3. The estimators of sequence diversity and richness. [file 12917_2020_2477_MOESM1_ESM.pdf]

Table S1: The information of sheep in this study.

No.	Breeds	Antibiotic treatment	Region	Health condition
1	Dorset sheep(DsS)	none	Qinghai Province, China	healthy
2	Dorset sheep(DsS)	none	Qinghai Province, China	healthy
3	Dorset sheep(DsS)	none	Qinghai Province, China	healthy
4	Dorset sheep(DsS)	none	Qinghai Province, China	healthy
5	Dorset sheep(DsS)	none	Qinghai Province, China	healthy
6	Dorset sheep(DsS)	none	Qinghai Province, China	healthy
7	Dorset sheep(DsS)	none	Qinghai Province, China	healthy
8	Dorset sheep(DsS)	none	Qinghai Province, China	healthy
9	Dorset sheep(DsS)	none	Qinghai Province, China	healthy
10	Dorset sheep(DsS)	none	Qinghai Province, China	healthy
11	Small Tail Han sheep(STHS)	none	Qinghai Province, China	healthy
12	Small Tail Han sheep(STHS)	none	Qinghai Province, China	healthy
13	Small Tail Han sheep(STHS)	none	Qinghai Province, China	healthy
14	Small Tail Han sheep(STHS)	none	Qinghai Province, China	healthy
15	Small Tail Han sheep(STHS)	none	Qinghai Province, China	healthy
16	Small Tail Han sheep(STHS)	none	Qinghai Province, China	healthy
17	Small Tail Han sheep(STHS)	none	Qinghai Province, China	healthy
18	Small Tail Han sheep(STHS)	none	Qinghai Province, China	healthy
19	Small Tail Han sheep(STHS)	none	Qinghai Province, China	healthy
20	Small Tail Han sheep(STHS)	none	Qinghai Province, China	healthy
21	Small Tail Han sheep(STHS)	none	Qinghai Province, China	healthy
22	Small Tail Han sheep(STHS)	none	Qinghai Province, China	healthy
23	Small Tail Han sheep(STHS)	none	Qinghai Province, China	healthy
24	Small Tail Han sheep(STHS)	none	Qinghai Province, China	healthy
25	Small Tail Han sheep(STHS)	none	Qinghai Province, China	healthy
26	Tibetan sheep(TS)	none	Qinghai Province, China	healthy
27	Tibetan sheep(TS)	none	Qinghai Province, China	healthy
28	Tibetan sheep(TS)	none	Qinghai Province, China	healthy
29	Tibetan sheep(TS)	none	Qinghai Province, China	healthy
30	Tibetan sheep(TS)	none	Qinghai Province, China	healthy
31	Dorper sheep(DrS)	none	Qinghai Province, China	healthy
32	Dorper sheep(DrS)	none	Qinghai Province, China	healthy
33	Dorper sheep(DrS)	none	Qinghai Province, China	healthy
34	Dorper sheep(DrS)	none	Qinghai Province, China	healthy
35	Dorper sheep(DrS)	none	Qinghai Province, China	healthy
36	Dorper sheep(DrS)	none	Qinghai Province, China	healthy
37	Dorper sheep(DrS)	none	Qinghai Province, China	healthy
38	Dorper sheep(DrS)	none	Qinghai Province, China	healthy
39	Dorper sheep(DrS)	none	Qinghai Province, China	healthy
40	Dorper sheep(DrS)	none	Qinghai Province, China	healthy

Table S2: Sequence data of samples.

Group	Raw sequences	High quality valid sequences	OTUs	Average valid sequences of sample
**DrS**	444002	355132	7039	35514
**DsS**	641786	507753	6887	33851
**TS**	203332	167035	4112	33407
**STHS**	405144	329485	8257	32949

Table S3: The estimators of sequence diversity and richness.

Sample ID	0.97			
	Simpson	Chao1	ACE	Shannon
DsS1	0.996	2723.14	2937.31	9.55
DsS2	0.997	3103.62	3327.68	9.9
DsS3	0.997	3439.89	3488.37	9.98
DsS4	0.997	2832.73	2905.67	9.71
DsS5	0.997	2385.47	2429.78	9.88
DsS6	0.998	3486.62	3701.14	10.19
DsS7	0.996	2691.09	2827.07	9.49
DsS8	0.998	3813.87	3798.53	10.25
DsS9	0.998	3631	3776.38	10.19
DsS10	0.997	3491.47	3434.29	10.09
STHS1	0.998	3629.71	3849.4	10.4
STHS2	0.998	3640.8	3639.86	10.4
STHS3	0.998	3488.14	3687.34	10.17
STHS4	0.998	3320.24	3442.62	10.21
STHS5	0.998	3621.41	3607.46	10.36
STHS6	0.998	2446.48	2459.22	10.2
STHS7	0.998	3567.37	3575.85	10.44
STHS8	0.997	2720.1	2858.37	9.99
STHS9	0.998	3521.68	3520.49	10.23
STHS10	0.997	3640.33	3765.47	10.11
STHS11	0.971	733.57	712.97	6.9
STHS12	0.975	1159.52	1106.44	7.44
STHS13	0.991	1620.74	1601.05	8.38
STHS14	0.984	1173	1173	7.96
STHS15	0.973	690.92	691.66	6.81
TS1	0.958	1138.99	1169.45	7.14
TS2	0.995	2407.35	2506.83	9.32
TS3	0.992	1649	1649.72	8.9
TS4	0.978	1574.28	1681.46	7.57
TS5	0.983	1156.66	1125.47	7.5
DrS1	0.989	1680.66	1748.88	8.34
DrS2	0.986	1007.79	1019.94	7.56

DrS3	0.983	1895.23	1914.18	8.45
DrS4	0.984	959.14	971.54	7.42
DrS5	0.994	2840.34	3021.31	9.43
DrS6	0.993	2206	2206	9.54
DrS7	0.997	3350.73	3344.35	9.97
DrS8	0.997	2853.69	3018.84	10.09
DrS9	0.998	2366.15	2372.82	10.02
DrS10	0.996	2907.86	3048.11	9.75
Mean ±SD	0.991 ±0.0095	2514.17 ±990.66	2577.91 ±1027.96	9.26 ±1.16
